# Multiple discrete soluble aggregates influence polyglutamine toxicity in a Huntington’s disease model system

**DOI:** 10.1038/srep34916

**Published:** 2016-10-10

**Authors:** Wen Xi, Xin Wang, Thomas M. Laue, Clyde L. Denis

**Affiliations:** 1 Department of Molecular, Cellular, and Biomedical Sciences, Rudman Hall, University of New Hampshire, NH 03824, Durham, USA.

## Abstract

Huntington’s disease (HD) results from expansions of polyglutamine stretches (polyQ) in the huntingtin protein (Htt) that promote protein aggregation, neurodegeneration, and death. Since the diversity and sizes of the soluble Htt-polyQ aggregates that have been linked to cytotoxicity are unknown, we investigated soluble Htt-polyQ aggregates using analytical ultracentrifugation. Soon after induction in a yeast HD model system, non-toxic Htt-25Q and cytotoxic Htt-103Q both formed soluble aggregates 29S to 200S in size. Because current models indicate that Htt-25Q does not form soluble aggregates, reevaluation of previous studies may be necessary. Only Htt-103Q aggregation behavior changed, however, with time. At 6 hr mid-sized aggregates (33S to 84S) and large aggregates (greater than 100S) became present while at 24 hr primarily only mid-sized aggregates (20S to 80S) existed. Multiple factors that decreased cytotoxicity of Htt-103Q (changing the length of or sequences adjacent to the polyQ, altering ploidy or chaperone dosage, or deleting anti-aging factors) altered the Htt-103Q aggregation pattern in which the suite of mid-sized aggregates at 6 hr were most correlative with cytotoxicity. Hence, the amelioration of HD and other neurodegenerative diseases may require increased attention to and discrimination of the dynamic alterations in soluble aggregation processes.

Huntington’s disease (HD) in humans is an incurable and lethal disease resulting from progressive neurodegeneration. Its effects (e.g., dementia) and lethality derive from a genetic expansion of a polyglutamine (polyQ) stretch in the huntingtin protein (Htt) that promotes misfolded protein aggregates within the cell^1^. These aggregates are linked to cytotoxicity, although the proximal causes for neuron damage and loss remain unknown^2^,^3^. Aggregate formation causes effects on transcription, protein folding, protein degradation, and/or mitochondrial function, among other processes^4^,^5^,^6^,^7^. Recent studies on HD and related neurodegenerative diseases suggest that it is the soluble aggregates that are most deleterious^8^,^9^,^10^,^11^,^12^,^13^.

Expansions of polyQ sequences are known to cause at least nine different human diseases^14^. HD results from the expansion of the polyQ segment of the Htt protein from 25Q to at least about 38Q. PolyQ expansions up to 103Q are common, and increased progression of the disease and cytotoxicity is associated with increasing lengths of the polyQ region. For all of these polyQ diseases, it is the aggregate formation that leads to the disease. Longer polyQ segments are conducive to β-sheet formation^15^,^16^,^17^ that are recognized by chaperone proteins as misfolded and produce the aggregates characteristic of the disease^5^,^18^,^19^. Other aggregate forming proteins can also become incorporated into Htt-polyQ aggregates^20^,^21^. The etiology of HD progression remains unclear^12^.

There are two major current gaps in knowledge concerning Htt-polyQ aggregate formation. First, there is no knowledge as to the actual diversity and sizes of Htt-polyQ aggregates. Typically, SDD-AGE, a crude filter assay, or native gel electrophoresis is used to monitor aggregation, all of which only demonstrate that an aggregate exists^20^,^22^,^23^,^24^,^25^,^26^,^27^. Soluble aggregates are often portrayed as amorphous aggregates for lack of proper experimental definition^11^,^15^. Alternatively, fluorescent microscopy is used to identify aggregate loci in a cell in which insoluble aggregates, but not soluble aggregates, are followed^18^. Second, because Htt-polyQ aggregates may be of a variety of sizes, the potential exists for the composition and abundance within these aggregates to change in regard to cytotoxicity and disease progression. Increased definition of these types of aggregates would be advantageous^28^.

We have consequently used the technique of analytical ultracentrifugation with fluorescent detection (AU-FDS)^29^,^30^ to identify Htt soluble aggregates using the yeast HD model system. AU-FDS allows the identification of the sizes and abundances of soluble macromolecular complexes ranging in sizes up to at least 1000S (about 160 MDa)^31^,^32^,^33^,^34^. It allows the extremely rapid and precise (at least an order of magnitude better than sucrose gradient analysis) determination of the size of protein complexes. Moreover, AUC analysis is typically conducted at 20 °C, a temperature closer to physiological conditions than other techniques conducted at 4 °C, and is conducted in physiological buffers without excess concentrations of sucrose, for example, which may affect the composition and/or structure of protein complexes.

Because we have previously demonstrated that AU-FDS allows specific detection, discrimination, size determination, and relative quantitation of all macromolecular complexes containing specific proteins^31^,^32^,^33^,^34^, we have determined which complexes Htt-polyQ proteins can form. The particular advantage of AU-FDS analysis is that it allows one to identify the complexes of a fluorescently tagged molecule (GFP in this case) in an impure mixture or cellular extract. Our AU-FDS analysis of Htt-tagged GFP molecules expressed in yeast^18^,^35^ indicates that initially upon expression both the non-toxic (Htt-25Q) and cytotoxic (Htt-103Q) Htt proteins formed soluble aggregates ranging in size from 29S to at least 200S. Since the current literature indicates that Htt-25Q does not aggregate^21^,^26^,^36^, a reassessment of prior Htt-polyQ studies may be required. In addition, we found that only the cytotoxic Htt-103Q evolved at later times to form significant abundances of mid-sized aggregates (20S to 80S in size) and large sized aggregates (greater than 80S in size). Alterations in factors that reduced Htt-polyQ-induced lethality, all affected the Htt-103Q aggregation distribution. The mid-size aggregates (33S to 84S) expressed at 6 hr after induction were most correlative with toxicity. Importantly, the progression in Htt-103Q aggregation behavior could be disrupted depending on when its expression was abrogated. These results indicate that AU-FDS analysis can discriminate between different types of soluble aggregates and that a better understanding of how to treat HD and other neurodegenerative diseases may derive from the study of the formation, regulation, and lethality of these truly dynamically varying and multitudinous number of different forms.

## Results

### Identification of Htt-polyQ aggregates using AU-FDS

To analyze Htt-polyQ aggregates in yeast, we used the well-characterized Htt-polyQ expression plasmids developed by several laboratories that induce cytotoxicity in yeast and display reduced or enhanced cell death when combined with defects in various chaperones and regulatory factors^18^,^22^,^23^,^24^,^35^,^37^. Htt-25Q is not toxic (summarized in Table 1) and does not form aggregates visible by microscopy (although some type of soluble aggregates of Htt-25Q are formed)^37^. In contrast, the Htt-103Q protein also forms insoluble aggregates visible by microscopy that may result in negative effects on yeast growth^18^,^22^. Htt-polyQ was expressed from a galactose promoter, as it allows specific induction of Htt-polyQ expression with the addition of galactose, thus avoiding maintenance of prior high levels of Htt-polyQ protein in cells that might lead to other confounding mutations^38^. In this case, growth on galactose medium resulted in Htt-103Q inhibiting cell growth as shown in Table 1: following one day of expression little cell growth was apparent for Htt-103Q relative to Htt-25Q and even after three days growth single colonies (third diluted pool in Table 1) were still particularly inhibited for Htt-103Q compared to Htt-25Q (also Supplementary Figure 1). The Htt-polyQ proteins also contained both a Flag N-terminal tag (allowing for a one-step pull down of Htt-containing complexes) and a GFP C-terminal attachment (allowing visualization of the complexes containing Htt-polyQ by AU-FDS).

**Figure 1:**
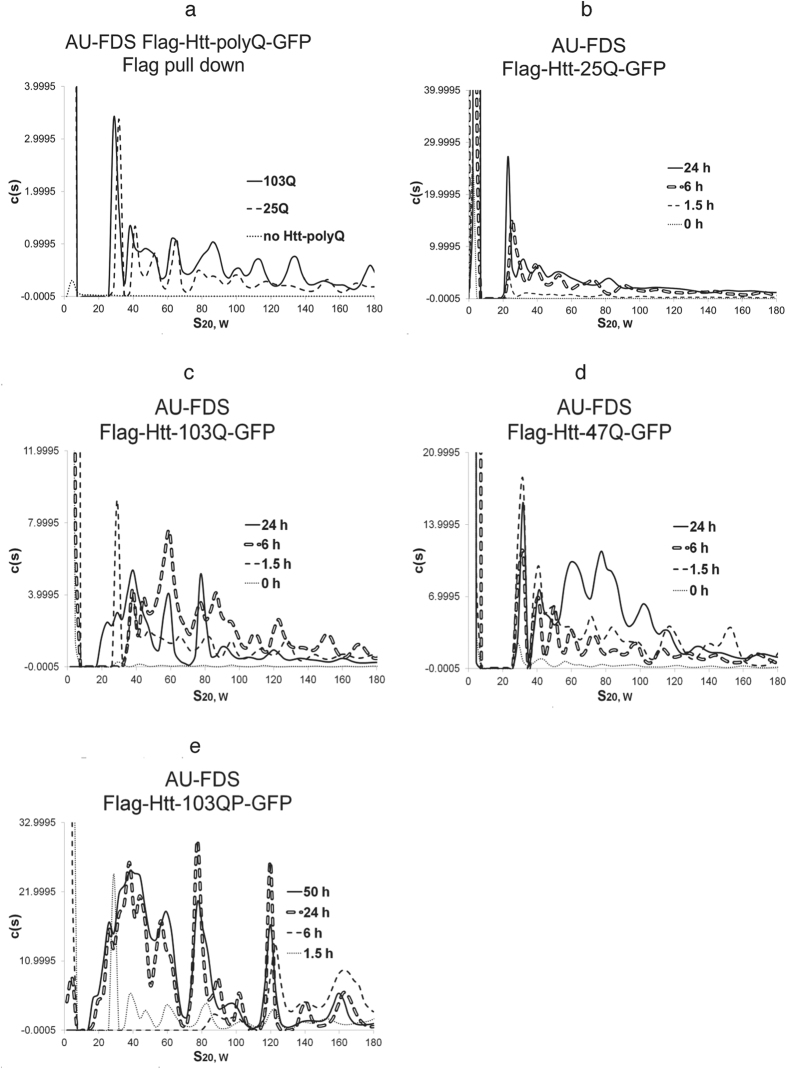
AU-FDS analysis of Htt-polyQ aggregation. Flag pull downs were done as described in Materials and Methods. Galactose induction of Htt-polyQ expression was done at the times indicated, except in panel ‘a’ induction was done for 1.5 hr. A rotor speed of 25K was used in all experiments. The diploid was formed by mating W303-1A to W303-1B. In a particular panel the absolute c(S) values can be relatively compared. However, c(S) values between panels can not be absolutely reliably compared due to possible alterations in the fluorescence optics of the AU-FDS instrument over time and due to differences in total number of cells harvested for each separate experiment. However, in general as shown in Supplementary Figure 2, different strains harvested at relatively the same mid-log growth stage expressed relatively similar levels of Htt-polyQ, except for that containing the *ssa1/ssa2* deletion as discussed in the text. (**a**) Htt-103Q, Htt-25Q, and no Htt. (**b**) Htt-25Q; (**c**) Htt-103Q; (**d**) Htt-47Q; and (**e**). Htt-103QP; and f. Diploid containing Htt-103Q.

As shown in Fig. 1a for a galactose induction time of 1.5 hr, following a one-step Flag pull down and AU-FDS analysis, Htt-25Q complexes ranging from 30S to larger than 180S were detected. Extracts from a control strain lacking Htt-polyQ and subjected to a Flag pull down did not display any of these complexes (Fig. 1a). While the major peak at 1.5 hr had an S coefficient of 30S, larger discrete complexes were detected with S coefficients of 40S, 49S, 60S, 71S, 86S, and larger values (average of three replicas with Standard Error of the Means, S.E.M.s, less than 1.5% for each peak). Htt-103Q at 1.5 hr post induction displayed the same general pattern as that of Htt-25Q with complexes with S coefficients of 29S, 39S, 48S, 59S, 70S, and 82 S (average of four to six replicas with S.E.M.s of less than 5%) (Fig. 1a). The intermediate Htt-47Q form that also displays intermediate cytotoxicity (Table 1) showed the same pattern of soluble complexes at 1.5 hr (Fig. 1d). Hence, soon after induction, both the non-toxic and toxic soluble aggregates of Htt-polyQ looked very similar. All of these Htt forms, prior to galactose induction, contained only limited material in complexes sized at 29S or larger (see Fig. 1b to 1d), indicating that the Htt complexes formed principally upon induction of expression with galactose. Both Htt-25Q and Htt-103Q displayed similar growth rates in liquid medium following induction with galactose: a 1.4-fold increase in cell numbers was observed at 1.5 hr, a 2.5-fold increase was observed at 6 hr, and about a 3-fold increase was observed by 24 hr.

All three forms of Htt-polyQ at 1.5 hr post induction contained sizeable amounts of material less than 10S (sized to be 2.5S for Htt-103Q, exactly in agreement with previous AU-FDS analysis and sizing of monomeric Htt-polyQ-GFP)^39^ (Fig. 1b to 1d). This material is indicative of monomeric proteins (if spherical, being of a mass of 22 KDa, correlating with the estimated size for each Htt-polyQ protein: 32 KDa, 35 KDa, and 42 KDa, respectively, for Htt-25Q, -47Q, and -103Q). Because the 29 to 30S complex formed with these Htt-polyQ proteins, if spherical, would be about 830 kDa in size (if elongated with a 2:1 length to width ratio, about 1.4-fold greater in size), then all complexes of 29S in size or larger appear to be aggregated forms of Htt-polyQ.

### Htt-103Q aggregation patterns change with cell growth

**Figure 2:**
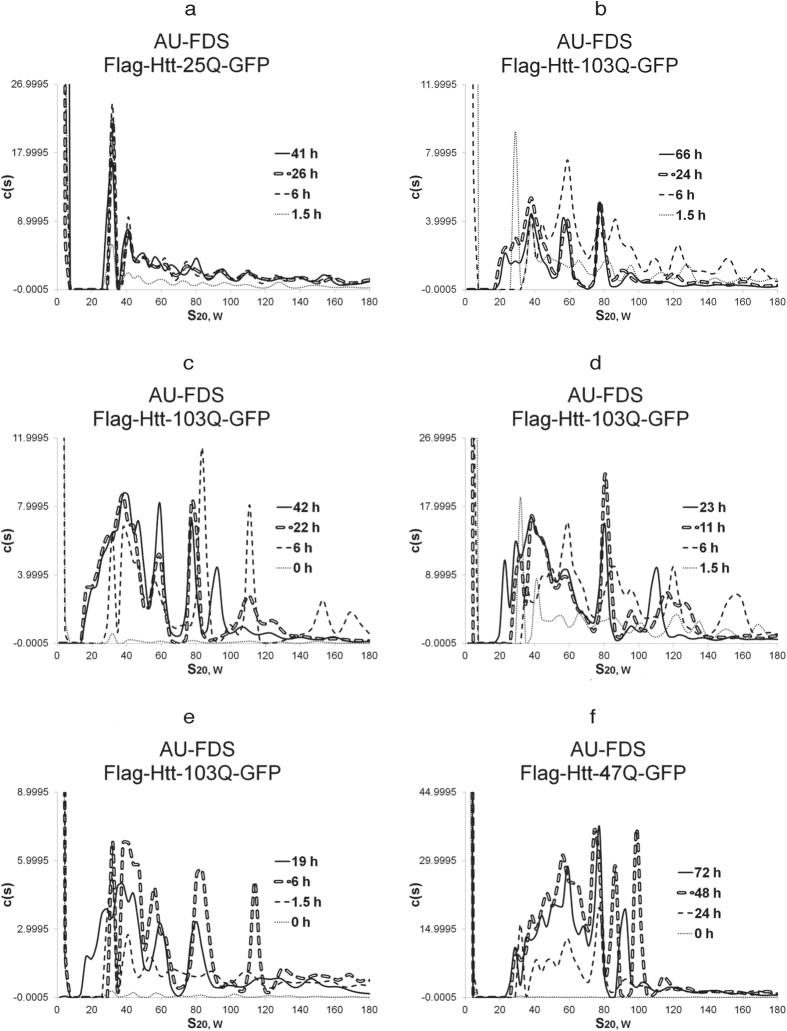
AU-FDS analysis of Htt-polyQ aggregation. All experiments were conducted as described in Fig. 1, and cell extracts were obtained at the time periods indicated. (**a**) HTT-25Q; (**b** to **e**) Htt-103Q; and (**f**) Htt-47Q.

We subsequently induced Htt-polyQ expression for times ranging up to 72 hr (Figs 1b–d and 2a–f). This extended analysis was conducted since the aggregation state and cytotoxicity of Htt-polyQ proteins and HD progression is known to change in respect to cell aging^22^. Htt-25Q did not show any significant shift in the pattern of soluble aggregates detected from 1.5 hr to 41 hr (Figs 1b and 2a), except more Htt-25Q material was being synthesized as the induction time increased.

Htt-103Q, however, displayed much different results (Fig. 1c). At 6 hr after induction, the 29S peak disappeared, and 33S to 84S peaks became more pronounced. For Htt-103Q, the patterns at 24 hr, 42 hr, and 66 hr were all similar to each other, and all showed significant differences as compared to that of the 1.5 hr and 6 hr times (Figs 1c and 2b to 2e). By 24 hr, new peaks at 20S were observed, a 28S peak reappeared, and peaks at 38S to 80S became predominant and remained so at later times. This appearance of 20S and 28S peaks was found to begin as early as 11 hr post-induction, but was not complete until 19 hr (Fig. 2d,e). The ratio of these mid-size peaks (20S to 80S) to larger ones (greater than 100S out to 250S) shifted during induction such that there was significantly much less of the larger aggregates as time progressed. For example, for 0 min this ratio was 1.1, for 1.5 hr it was 1.2, for 6 hr 1.7, and for 24, 42, and 66 hr it was about 3.4. Therefore, at late times more mid-size complexes were formed relative to the large complexes. Importantly, all of the monomeric proteins nearly completely disappeared sometime between 11 hr and 19 hr (Figs 1c and 2d,e), suggesting that monomers had become fixed into Htt-103Q aggregates at late time points. It should also be noted that in addition to the S coefficient values being highly reproducible between experiments, the peak intensities within profiles remained very similar between experiments. (e.g., compare the 19 hr to 24 hr profiles of Htt-103Q in Fig. 2b to 2e).

The pattern of soluble aggregates for Htt-47Q also changed during later periods of induction, but the results both were not as dramatic as that observed for Htt-103Q and lacked certain features (Figs 1d and 2f). At 6 hr after induction, Htt-47Q displayed the same general aggregation pattern as it did at 1.5 hr, similar to that which was observed for Htt-25Q. However, by 24 hr, an increase in abundance of mid-sized complexes ranging in size between 30S and 80S occurred (Fig. 1d). At later time, these mid-sized peaks, while increasing in abundance from 24 hr to 47 hr, did not shift in size distribution out to 72 hr (Fig. 2f). It should also be observed that, unlike Htt-103Q, there was no loss of the 28S complex or shift to slightly smaller sized complexes (20S) at later times after induction for Htt-47Q. Little large sized complexes (greater than 100S) were observed for Htt-47Q, and the monomer pool did not disappear at 24 hr or later times, as it had for Htt-103Q. Therefore, Htt-47Q, which displays an intermediate level of toxicity (Table 1), results in a both a delayed formation of the mid-sized aggregates at 6 hr that are abundantly present in the extremely toxic Htt-103Q form and fails to form the large aggregates that are principally formed at 6 hr with Htt-103Q.

### The proline-rich region of Htt that abrogates toxicity reduces 6 hr mid-sized Htt-103Q aggregation as do changes in ploidy

To clarify which of the soluble aggregate sizes observed with Htt-103Q were correlated with toxicity as observed in our plate assay, we subsequently conducted a time course of induction on Flag-Htt-103Q-PRO-GFP (Htt-103QP). The presence of the proline-rich region is known to block cytotoxicity of Htt-103Q (Table 1)^23^,^24^. As shown in Fig. 1e, at 1.5 hr the pattern of aggregates for Htt-103QP was found to be very similar to that observed for all of the Htt-polyQ variants at 1.5 hr. However, at 6 hr after induction of Htt-103QP, only large soluble aggregates were present (greater than 80S), and it was not until 24 hr that the medium-sized aggregates appeared that are characteristic of Htt-103Q. In contrast to Htt-103Q, the large sized aggregates of Htt-103QP ranging from 100S to 250S remained predominate even at times out to 50 hr. Monomers were also shown to disappear at 24 hr, as was observed for Htt-103Q. These results correlate the absence of medium-sized aggregates at 6 hr with the previous observed lack of lethality at 8 hr for Htt-103QP^23^,^24^. The observation that at times after 6 hr for Htt-103QP the mid-sized aggregates were abundantly present and the large sized aggregates remained as well suggests that the lethality with Htt-103Q is neither correlated with the presence of these mid-sized aggregates at late times nor with the presence of large sized aggregates at 6 hr or later. Rather, the lack of lethality of Htt-103QP (and the reduced toxicity of Htt-47Q) may be directly related to the reduced formation of a large pool of mid-sized aggregates early in the induction process at 6 hr. These results imply a time-dependent effect in regards to Htt-103Q-induced lethality or specific features of the 6 hr aggregates formed with Htt-103Q.

**Figure 3:**
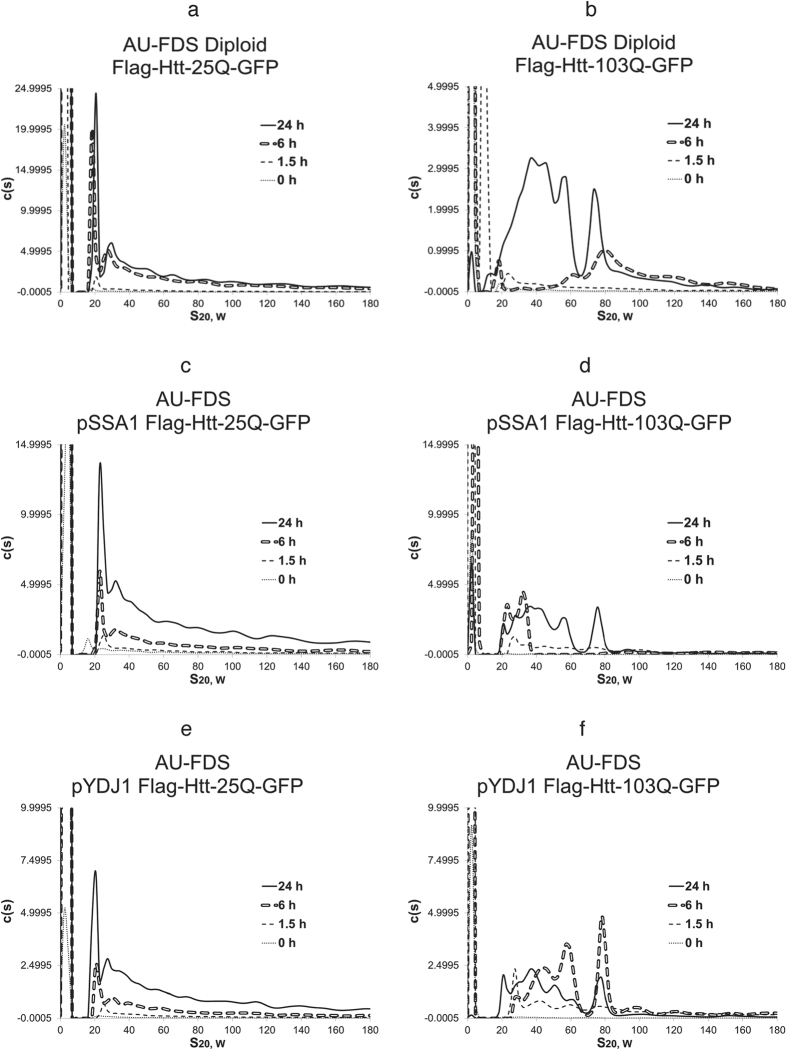
AU-FDS analysis of Htt-polyQ aggregation as affected by alterations in ploidy and chaperone dosage. All experiments were conducted as described in Fig. 1. a, c, and e. Htt-25Q; and b, d, and f. Htt-103Q. (**a**,**b**). Diploid; (**c**,**d**). Overexpression of *SSA1* with plasmid pSSA1; and (**e**,**f**). Overexpression of *YDJ1* with plasmid pYDJ1.

Ploidy effects on Htt-polyQ lethality have been observed previously^25^, and we showed that Htt-103Q displays reduced lethality in diploids (Table 1). In diploids, Htt-25Q displayed the same relative pattern of aggregated Htt proteins as was shown for haploid cells (Fig. 3a). However, in diploids, Htt-103Q aggregation pattern was markedly altered (Fig. 3b). The Htt-103Q expression pattern at 6 hr Htt-103Q failed to develop the usual pattern of mid-sized aggregates that ranged from 33S to 84S in size. Large sized aggregates and only some 80S complexes were formed. By 24 hr, the Htt-103Q pattern reverted to being most similar to the 24 hr pattern for Htt-103Q. These occurrences support the previous observation that the lethality of Htt-103Q inheres to differences in aggregation patterns at 6 hr in which a broad range of mid-sized aggregates are being formed.

### Deletions of the *HSP70 and HSP104* chaperone genes rescue Htt-103Q-induced lethality and alter its aggregation patterns

**Figure 4:**
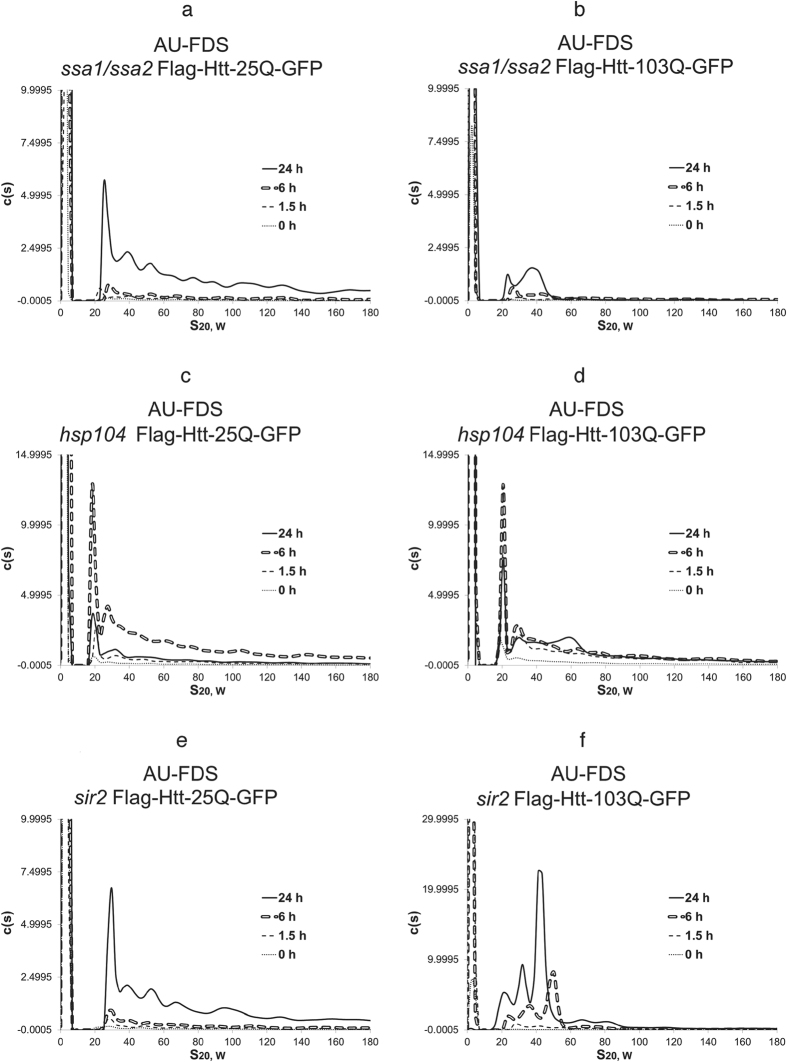
The effects of deleting chaperones and *SIR2* on Htt-polyQ aggregation. AU-FDS analysis was conducted as described in Fig. 1 in strains carrying Htt-25Q (**a**,**c**,**e**) and Htt-103Q (**b**,**d**,**f**). (**a**,**b)** *ssa1/ssa2*; (**c**,**d**). *hsp104*; and (**e**,**f**) *sir2*.

We subsequently investigated the effects of altering the dosage of chaperones known to affect Htt-103Q toxicity^18^,^37^,^40^. We initiated these analyses with a strain carrying a deletion of the *SSA1*/*SSA2* genes, encoding for the HSP70 chaperones. The *ssa1*/*ssa2* deletion is known to suppress Htt-103Q lethality and to affect insoluble aggregation as detected by fluorescent microscopy (Table 1)^35^. We also found that deleting the *SSA1* and *SSA2* genes decreased the growth rate of cells expressing Htt-25Q (Table 1), implying that these deletions were exerting other pleiotropic effects on the cell. When Htt-25Q and Htt-103Q were expressed in cells lacking SSA1/SSA2, several effects were observed. First, the overall fluorescent signal that was obtained for either Htt-25Q or Htt-103Q at 1.5 hr post induction was not significantly above the non-induced level of expression (compare Fig. 4a,b, respectively, to Fig. 1b,c). Second, by 24 hr the Htt-25Q pattern in the *ssa1*/*ssa2* deletion strain looked very similar to that of Htt-25Q expressed in the wild-type strain. Third, the Htt-103Q pattern of aggregation at 6 hr in the *ssa1*/*ssa2* background lacked appreciable mid-sized or larger aggregates, and at 24 hr most mid-sized complexes and all large sized aggregates were absent (Fig. 4b). Fourth, the monomeric form of Htt-103Q did not diminish at 24 hr, implying either delayed incorporation of monomers into larger sized aggregates or, if insoluble aggregates are forming, then enhanced breakdown of these into constituent monomers. These observations indicate that removal of the SSA1 and SSA2 chaperones from the cell profoundly alters Htt-103Q soluble aggregation behavior that is observed at 6 hr. These effects are correlative with the reduction in the Htt-103Q lethality that deleting *SSA1/SSA2* causes.

Similar analyses were conducted with a strain carrying the *hsp104* deletion, which has been shown to block toxicity (Table 1) and insoluble aggregation^18^,^37^. HSP104 functions as a disaggregase that may promote cell lethality by creating smaller aggregates that can reseed in cells^41^,^42^. In the *hsp104* deletion strain, Htt-25Q displayed normal expression of soluble aggregates at 6 hr post-induction but vastly diminished levels at 24 hr post induction (Fig. 4c). This result implies that HSP104 is required for either stable formation or accumulation of Htt-25Q aggregates at late times.

Importantly, the *hsp104* deletion displayed strong effects on the Htt-103Q lethality and aggregation pattern. This deletion suppressed increased expression of Htt-103Q after 1.5 hr, blocked at 6 hr both mid-sized and large sized aggregate formation, and resulted at 24 hr in a pattern similar to the control Htt-25Q in the wild-type strain at all times or Htt-103Q after 1.5 hr of induction (Fig. 4d). The *hsp104*-induced reduction in total aggregation of Htt-25Q at 24 hr compared to that of 6 hr observed for Htt-25Q was not as dramatic for Htt-103Q but was observed. It should be noted that the *hsp104* deletion did not reduce the growth of these cells in liquid medium as compared to the wild-type cells, indicating that these abundance changes observed for the *hsp104* deletion at late times result from intracellular effects on expression and/or aggregation of the Htt-polyQ proteins. Finally, for the *hsp104* deletion, monomeric Htt-103Q protein did not diminish at 24 hr, a result that was similar to that observed for the *ssa1*/*ssa2* deletion. Overall, these results again support a strong correlation between the ability of Htt-103Q to form soluble mid-sized aggregates at 6 hr and the ability of Htt-103Q to cause cytotoxicity.

### SSA1 overexpression blocks Htt-103Q soluble aggregation patterns and its lethal effects

Strains overexpressing HSP70 (using the pSSA1 plasmid) were also analyzed in which overexpression had no significant effects on Htt-25Q aggregation (Fig. 3c). Overexpression of SSA1, in contrast, significantly affected the aggregation patterns observed with the Htt-103Q (Fig. 3d). At 1.5 hr there was a diminution in the formation of the 29S complex and at 6 hr a total blockage in formation of mid-sized complexes larger than 35S and also of that of large sized aggregates. By 24 hr the mid-sized aggregates were observed that are typically found with Htt-103Q, confirming that their presence at 24 hr is not correlated with lethality. The monomeric form also became reduced at 24 hr, indicative of its successful incorporation into the mid-sized aggregates. The appearance of 20S-sized aggregates observed with Htt-103Q in the wild-type strain was also observed in the presence of the overexpressed form of SSA1. As overexpression of *HSP70* suppresses the toxicity of Htt-polyQ (Table 1)^43^,^44^, these results suggest the reduction in the formation of 40S to 80S mid-sized at 6 hr with overexpressed SSA1 is linked to preventing cell death.

As a control, we also analyzed the effect of overexpression of *YDJ1*, a member of the HSP40 chaperone family. The co-chaperone YDJ1 interacts with the HSP70 chaperones to regulate their activity, and overexpression of HSP40 reduces the size and amount of amyloids formed in different neurodegenerative diseases^43^,^44^. However, overexpression of *YDJ1* does not significantly suppress Htt-103Q toxicity in yeast^18^. Although a very modest suppression of overexpression of *YDJ1* on Htt-103Q toxicity was observed in our strains, further analysis verified that the strain Htt-103Q in a strain containing overexpressed*YDJ1* exhibited complete lethality, as did the wild-type strain containing Htt-103Q (Table 1). No effect of *YDJ1* overexpression was observed on Htt-25Q aggregation (Fig. 3e). In terms of Htt-103Q aggregation, overexpression of *YDJ1* resulted in a set of peaks more similar to those observed in the wild-type strain, except that overall expression of Htt-103Q was reduced and the formation of large sized aggregates observed at 6 hr did not occur (Fig. 3f). At 24 hr*YDJ1* overexpression did not decrease monomer incorporation into aggregates and the mid-sized aggregate pattern was similar to that observed in the wild-type strain with the usual appearance of aggregates of 20S in size. These results imply that overexpression of *YDJ1* is having more modest effects on Htt-103Q soluble aggregate formation and that reductions in large sized aggregation at 6 hr do not correlate with lethality.

### Deletion of *sir2* affects the Htt-103Q aggregation pattern and overcomes Htt-103Q lethality

Based on the above results, we subsequently analyzed the effects of deleting the *SIR2* gene on Htt-polyQ aggregation. SIR2 is an anti-aging factor, orthologous to mammalian Sirt 1 that has been linked to affecting neurodegeneration^45^. The deletion of *sir2* was found to promote the formation of insoluble aggregates of Htt-103Q in yeast cells^22^, and we showed that it blocked the lethality associated with Htt-103Q (Table 1). In correlation with these effects on lethality of Htt-103Q, deletion of *SIR2* dramatically changed the pattern of soluble aggregate complexes found with Htt-103Q while the Htt-25Q aggregates remained unaffected (Fig. 4f,e, respectively). For Htt-103Q at the 6 hr time point, only three major complexes migrating between 20S and 50S were observed in the AU-FDS results. None of the larger 80-180S complexes were observed that was present in the wild-type yeast strain. At 24 hours, the three complexes were again observed but each was of slightly reduced S value. The typical appearance of slightly smaller complexes of about 20S that occurred with Htt-103Q in the wild-type strain at later times was observed early at 6 hr in the *sir2* background. The decrease in the levels of the monomeric form also occurred at 24 hr for Htt-103Q. The overall pattern of changes in Htt-103Q aggregates with the *sir2* deletion suggests that interfering with the formation of specific mid-sized aggregates (60S to 80S), and perhaps the pattern of other mid-sized aggregates, were correlative with the effect of this deletion on overcoming Htt-103Q-induced cytotoxicity.

### Analysis of the ability of yeast to clear soluble aggregates from the cell

**Figure 5:**
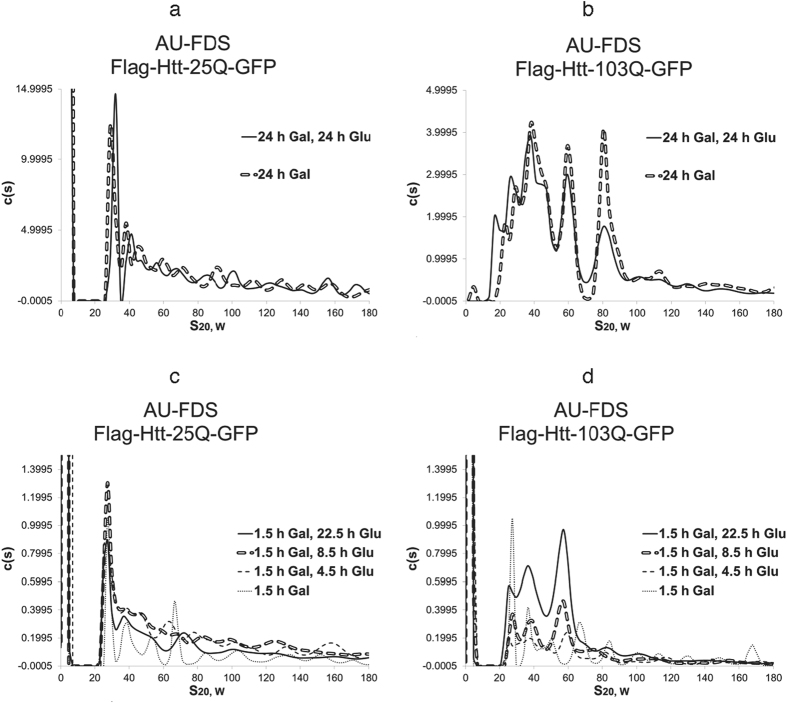
Changes in Htt-polyQ aggregation following cessation of synthesis. AU-FDS analysis was conducted as described in Fig. 1 in strains carrying either Htt-25Q (**a**,**c**) or Htt-103Q (**b**,**d**). For panels (**a**,**b**) strains were induced for Htt-polyQ expression for 24 hr with galactose at which times 2% glucose was added; (**c**,**d**) strains were induced for Htt-polyQ expression for 1.5 hr with galactose at which times 8% glucose was added The higher concentration of glucose was used to ensure complete shut off of the *GAL1* promoter.

The above results indicate that by at least 24 hr post-induction Htt-103Q settles into an aggregation pattern that is extremely stable in the yeast cell. We consequently analyzed whether this stability was due to inability of the yeast to alter aggregated Htt-103Q molecules once formed or the result of continual new synthesis of aggregates that replenished recycled aggregates. To approach this problem, we shut off Htt-103Q *GAL1*-induced expression with the addition of glucose 24 hr post-induction. We then monitored at 24 hr after repression of Htt-polyQ expression the pattern of Htt-polyQ aggregation. For Htt-25Q, shutting off for 24 hr the expression of the *GAL1* promoter did not result in any appreciable diminution or alteration in the pattern of soluble aggregates (Fig. 5a). The same result was obtained with Htt-103Q (Fig. 5b). Hence, once these terminal soluble aggregates are formed within yeast cells, the yeast cell is unable to clear or otherwise alter their presence.

Because the 6 hr soluble aggregation pattern of Htt-103Q appears particularly correlated with cell lethality, we subsequently tested whether shutting off of Htt-polyQ expression prior to this time period would alter Htt-polyQ aggregation behavior or allow the cell to clear the aggregates. In this situation, we added glucose to cells that had only been induced for Htt-polyQ expression for only 1.5 hr with galactose. Both Htt-25Q and Htt-103Q display very similar aggregation patterns at 1.5 hr post-galactose induction (Fig. 1a), but after this time only Htt-103Q develops significant alterations in the aggregation pattern (Fig. 1c). As expected, Htt-25Q displayed very similar aggregation patterns out to 22.5 hr after glucose repressed its expression as compared to those observed at 1.5 hr post-galactose induction (Fig. 5c), indicating that once the initial Htt-25Q aggregates are formed they do not change significantly in abundance or size and the cell can not alter their structure.

In contrast to Htt-25Q, Htt-103Q aggregation behavior continued to develop although the expression of Htt-103Q was shut off (Fig. 5d). At 4.5 hr after shut off of Htt-103Q expression, the 29S complex remained (whereas it disappeared when expression was not shut off), and only mid-sized aggregates out to 60S were predominantly formed. At 8.5 hr after glucose addition, three major peaks migrating between 29S and 58S were predominant and remained so at the later time of 22.5 hr after promoter shut-off. Hence, once formed the Htt-103Q aggregates cannot be cleared from the cell and instead they develop in a yeast cell into at least three mid-sized aggregates similar to those observed under normal induction conditions with Htt-103Q: about 29S, 37S, and 58S in size. Yet, these patterns were distinct from the normal aggregation pattern of Htt-103Q when expression is not ceased, with especially little or no formation of the 84S complex that appeared to be linked to cell lethality, no loss of the 29S complex 6 hr after induction, and no appearance of 20S complexes at later times. These observations imply that yeast may be able to accomplish some alterations in Htt-103Q aggregation behavior if Htt-polyQ expression can be shut off at an appropriately early time point before the panoply of lethal Htt-103Q aggregates are formed. It should also be noted that the abundance of these three mid-sized aggregates increased even though expression had been shut off with a very high percentage of glucose. Because monomer abundance did not change significantly between 4.5 hr after repression out to 22.5 after repression, there appears to be no new synthesis of monomers or significant depletion of monomers as in their incorporation into the mid-sized aggregates observed. It is possible, therefore, that the increases in the abundance of the these mid-sized aggregates that are being observed result from contributions from insoluble aggregated material not observed in these experimental conditions.

## Discussion

We used AU-FDS to identify for the first time a diverse group of discrete soluble aggregates that polyQ-containing proteins can form. We find for the non-toxic Htt-25Q that it forms primarily a 30S complex, but aggregates up to at least 200S were identified. The 30S complex, if spherical would be 0.83 MDa in size, but could be much larger if elongated. As the Htt-25Q protein is only 32 KDa, these aggregates imply significant polymerization of the protein, although other proteins such as chaperones and mis-incorporated would be expected to associate with the Htt-25Q protein^19^. During the induction of Htt-25Q this pattern of aggregate formation did not change, confirming these particular aggregates as not toxic to the cell. The fact that Htt-25Q can form soluble aggregates, albeit not detected by SDD-AGE or other methods used previously to study Htt aggregates, indicates an inherent capability of even short polyQ regions to aggregate. Therefore, our results indicate it is not a question of whether short or long polyQ sequences allow aggregation, but what type of aggregates can be formed from a particular length of polyQ sequence.

Importantly, the fact that Htt-25Q can form a range of aggregate complexes extending to the truly large (200S) indicates that current models and studies of Htt-polyQ aggregation are incomplete and need reevaluation. While oligomeric forms of Htt-polyQ are assumed to exist, such terminology does not adequately express what is present in the cell. Importantly, several previous mass spectrometric studies on polyQ aggregates relied on using proteins with short polyQ sequences as controls to eliminate supposedly contaminating proteins detected with the analysis^5^,^21^. Our results indicate that because Htt-25Q forms very large aggregates, using proteins such as Htt-25Q as controls would have led to these analyses eliminating a number of proteins that do associate with polyQ aggregates. These missed proteins (found associating with both short and long polyQ aggregates) would be expected to contribute significantly to aggregation formation, behavior, and lethality.

In terms of the Htt-103Q protein, after 1.5 hr of induction it formed the exact same pattern of aggregates that Htt-25Q formed at all times after induction. Htt-47Q behaved the same. This similarity in pattern implies that these aggregates at 1.5 hr are the initial forms that can be created by associating Htt-polyQ proteins irrespective of the polyQ length. However, the aggregate pattern for Htt-103Q evolved at later times in which four specific pronounced changes were identified. First, mid-sized aggregates 33S to 84S and large sized aggregates greater than 100S became predominant at 6 hr. Second, by 24 hr and later times, the large sized aggregates diminished in abundance and the mid-sized aggregates were the predominant species. Third, the smallest aggregate of 29S disappeared at 6 hr and by 24 hr aggregates migrating at 20S to 28S reemerged. Fourth, the monomeric form disappeared at 24 hr, implying incorporation of almost all monomers into the above pattern of aggregates.

Correlation between the above patterns of aggregation for Htt-25Q and Htt-103Q and cytotoxicity result in several principal observations. It is clear that the pattern inherent in Htt-25Q at all times and with Htt-103Q at 1.5 hr is not associated with lethality. However, lethality was associated with particular aggregation behavior of Htt-103Q at 6 hr. The presence of mid-sized aggregates (33S to 84S and, more specifically, the 50S to 84S group) were most correlative with cell lethality. Multiple observations support this conclusion. Htt-47Q displayed reduced cell lethality and did not form the 6 hr mid-sized aggregates that Htt-103Q did. The fact that the Htt-47Q pattern at 24 hr was most similar to that of the pattern found with Htt-103Q at 6 hr suggests that the aggregation patterns at 6 hr are tied to lethality, not necessarily those at 24 hr. Similarly, Htt-103QP was non-toxic and failed to form mid-sized aggregates at 6 hr, although, once again, its 24 hr aggregation pattern was nearly identical to that of Htt-103Q. Diploids carrying Htt-103Q also did not display cell death and were deficient in the 6 hr aggregation pattern characteristic of Htt-103Q but not that of the 24 hr pattern. Altering chaperone dosage or deleting the *SIR2* gene, effects that compromised cell lethality of Htt-103Q, correspondingly eliminated or severely altered the mid-sized aggregation patterns of Htt-103Q. In these latter cases (specifically overexpression of *SSA1* or deletion of *SIR2*), there was specific depletion of the 60S to 80S mid-sized aggregates. These latter results suggest that only some of the mid-sized aggregates present at 6 hr may be specifically linked to cell death. These observations suggest further that lethality of Htt-103Q may be tied to specific types of soluble aggregates formed at specific times following their initial expression. When a particular aggregate is formed may interfere with certain biological processes as related to cell lethality, an issue that has important implications for the etiology of HD and other aggregate diseases and their progression.

It is important to note, however, that for each of these Htt-103Q mid-sized aggregates present at 6 hr there is no implication that they are of the same type or composition of the same sized aggregates that are present at 24 hr. That is, the 6 hr mid-sized aggregates that are correlated with lethality may be of a different type and structure than the same sized aggregates observed at 24 hr. Hence, while the correlations between lethality and the 6 hr Htt-103Q aggregation pattern do suggest a time-dependent effect, such a correlation could be simply the result of a particular type of mid-sized aggregate formed at 6 hr that is not present at 24 hr. In either case, this possible change in form of similarly sized aggregates suggests an extremely dynamic process for aggregation. Such alterations would be expected to result in complex interactions with other cellular factors, as has been abundantly verified in a variety of aggregation-induced neurodegenerative diseases^12^. Based on the truly multitudinous forms we find and the distinct possibility that different forms can affect distinctly different cellular processes, the path to cell lethality may be far from monolithic, linear, and easily resolved based on whether aggregates are present or not. This observation may explain why polyQ toxicity has been linked to a plethora of cellular processes. Our identification of discretely sized soluble aggregates that occur at specific times during growth as linked to cell death may be useful to alleviating this impasse.

We also investigated the sensitivity of the Htt-polyQ aggregation behavior to cell clearance once gene expression ceased. Our results indicate that the cell is unable to clear such aggregates. This is especially true at 24 hr post-induction, implicating an implacable progression of aggregate formation that once begun could lead to disease progression. However, early in yeast expression of Htt-103Q some significant changes in aggregation behavior were observed if expression were ceased at 1.5 hr. Three major effects of this early intervention were observed. First, the 84S Htt-103Q aggregate, linked to cytotoxicity, did not form. Second, the 20S aggregates that are normally observed after 6 hr of induction did not form. Third, monomer incorporation into aggregates remained incomplete. These observations suggest that it may be possible to interfere with Htt-polyQ aggregate formation and perhaps HD progression by several means, including that of timely intervention in reducing Htt-polyQ expression.

Critical to Htt-103Q lethality was chaperone function. Deleting the HSP70 chaperones *SSA1/SSA2* or deleting *HSP104* affected Htt-103Q aggregation behavior and cell lethality was abrogated. For *ssa1*/*ssa2*, this deletion’s blocking of Htt-103Q toxicity appeared correlative to lack of formation of a specific subset of mid-sized aggregates (50S to 80S). In contrast, for *hsp104*, the resultant pattern of Htt-103Q aggregation distribution at all times became most similar to that of Htt-25Q, which failed to display any of the Htt-103Q aggregation changes outlined above. These varying effects imply that these different deletions individually are acting by different mechanisms on aggregates to affect cell toxicity and Htt-103Q aggregation. Increasing SSA1 abundance in the cell, which interfered with Htt-103Q toxicity, also blocked large sized and most mid-sized aggregate formation at 6 hr, although it did not appreciably interfere with the formation of the mid-sized aggregates at 24 hr or the incorporation of monomers into these aggregates. Altering *YDJ1* dosage, in contrast, did not appreciably interfere with cell lethality or aggregate formation. The ability of individual chaperones to target aggregates by distinct mechanisms and to alter aggregation behaviors in distinct ways support chaperones as a primary means by which aggregate patterns and behaviors may be influenced in the cell.

We also found that deleting the anti-aging gene *SIR2* had profound effects on Htt-103Q aggregate formation. The *sir2* deletion, which reduced cytotoxicity appeared to be having its major effects on the inability of forming aggregates larger than 50S and the rapid formation of only some mid-sized aggregates, conclusions consonant with those obtained by altering chaperone dosage. How the SIR2 protein leads to cell lethality in the presence of Htt-103Q remains unclear, however.

The ability of AU-FDS to differentiate these hitherto unknown patterns of change inherent in the Htt-103Q soluble aggregation process, indicates that more careful analysis of aggregation needs to be incorporated into the study of neurodegenerative diseases. Determining the composition of factors and their relative stoichiometry in each of the complexes we have identified would be an initial step to clarifying which aggregates affect which particular cellular processes. Moreover, AU-FDS identification of these complexes promises a more systematic evaluation of the efficacy of various proposed palliative HD treatments on actual aggregation phenomenon^4^,^46^,^47^.

## Materials and Methods

### Yeast strains, growth conditions, and extract preparation

Yeast strains were isogenic to W303-1A (*Mata leu2-3, 112 trp1-1 ura3-1 his3-11 ade2-1 can1-100*) (Table 2). The Flag-Htt-polyQ-GFP-containing plasmids were obtained from M. Sherman^35^ and contain Htt-polyQ expressed in the pYES2 vector. Overexpressed versions of *SSA1* (plasmid pLH101, pSSA1) and *YDJ1* (plasmid pRSYDJ1, pYDJ1) have been previously described^18^. For confirming cell lethality of Htt-polyQ expression, cells were streaked to singles either on selective medium containing 2% glucose or containing 2% galactose (described in detail in Table 1 and an example of which is displayed in Supplementary Figure 1). No differences in cell lethality for particular Htt-polyQ proteins were observed as compared to the published results (when available) for the strains used in this study (Table 1). The expression levels of Htt-103Q in the various strains were fairly comparable as shown in Supplementary Figure 2 when the monomeric form of Htt-103Q was followed by AU-FDS analysis, except for the strain containing the *ssa1/ssa2* deletion. In this latter case, this deletion resulted in reduced expression of Htt-103Q. The Flag-Htt-polyQ-GFP proteins expressed in yeast have been shown to result in toxic and cellular effects similar to those for Htt-polyQ expressed in mammalian model systems^18^,^22^,^23^,^24^,^35^. Moreover, mass spectrometric analyses identifying the proteins associating with the same Flag-Htt-103Q-GFP protein used in these studies found similar proteins as that associate with Htt-103Q in mammalian systems^21^,^48^. These correspondences suggest that the Flag and GFP tags are not affecting the Htt-polyQ aggregation phenomenon.

For cell extract preparation, yeast cells were grown at 30 °C in glucose-free synthetic complete (SC) medium until mid-log phase (OD600 = 0.8–1.8), with 1% sucrose, 1% raffinose and with the appropriate deficiency in amino acids for plasmid selection^18^. To induce the production of Htt-polyQ proteins, galactose was added to a final concentration of 2%. The incubation was continued for the designated period of time and cell pellets were collected by centrifugation at 8000 *g* for 3 min and stored at 80 °C. For each AU-FDS sample, 100 mL of yeast culture were collected, regardless of the final cell concentration at the time of collection. The protein content of yeast cells was extracted by the glass bead method with cell lysis buffer containing 1mg/mL of PMSF, 1:500 dilution of Protease Inhibitor Cocktail (Sigma-Aldrich P8215), 0.05 M of Tris Base, 0.15 M of KCl, 2 mM of MgCl_2_ and 10% glycerol. The Flag-tag complexes were purified by affinity purification with 100 μL of ANTI-FLAG^®^ M2 Affinity Gel (Sigma-Aldrich) at 4 °C for 2 hours, followed by a wash with cell lysis buffer without the Protease Inhibitor Cocktail. The Flag-associated complexes were eluted with 500 μL of 0.2 mg/mL FLAG^®^ Peptide (Sigma-Aldrich) at 4 °C for 40 minutes.

### AU analysis

Samples were loaded to the two-channel charcoal/Epon60K sedimentation velocity cells, with approximately 350 μL per channel. Centrifugation was conducted at a speed of 25000 RPM and a temperature of 20 °C. For the AU-FDS analysis, at least 200 scans of samples were taken from each experiment, in about 5 hours, a period of time sufficient for the major concentration boundary to migrate to the bottom of the cell. The raw data was analyzed by Sedfit^31^, with the model c(s) distribution and proper parameters. The calculated data set of S-values was adjusted by multiplying the viscosity factor of 1.52 for the presence of 10% glycerol in the buffer. Examples of the raw data for the AU-FDS analysis are presented in Supplementary Figure 3. At 24 hr for the Htt-103Q sample (AUC data displayed in Fig. 1c) as shown in Supplementary Figure 3a, top panel, discrete peaks are being visualized. This is seen in the later scans (right side of Figure) in which there are multiple shoulders present (darkened areas that run horizontally left to right) that are indicative of banding of multiple species. In the bottom panel for 24 hr, what are being shown on the y-axis are the residuals (differences between raw data and the fit curves). If there were only systematic noise, one should obtain a completely random distribution. As shown in this panel, after the first 10 or so scans (the extreme left of figure), the data randomly varies around the zero value. This indicates that there is essentially a random distribution out to at least 200 scans, indicating that Sedfit is not generating false peaks. Similar plots are presented for the Htt-103Q at 6 hr with similar conclusions, except the first 20 to 40 scans are not as good as they were for the 24 hr time point. After these number of scans the fit curves demonstrate random distributions.

## Additional Information

**How to cite this article**: Xi, W. *et al*. Multiple discrete soluble aggregates influence polyglutamine toxicity in a Huntington's disease model system. *Sci. Rep.* **6**, 34916; doi: 10.1038/srep34916 (2016).

## Supplementary Material

Supplementary Information

## Figures and Tables

**Table 1:** Effects of Htt-polyQ on cell growth.

Htt-polyQ	Relevent genotype	Growth on glc, 1 day	Growth on gal, 1 day	Growth on gal, 2 days	Growth on gal, 3 days
Htt-25Q	Wild-type	**+ + +**	**+ w −**	**+ + +**	**+ + +**
Htt-47Q	Wild-type	**+ + +**	**+ w− −**	**+ + −**	**+ + w**
Htt-103Q	Wild-type	**+ + +**	**w− − −**	**+ − −**	**+ w −**
Htt-103QP	Wild-type	**+ + +**	**+ w− −**	**+ + +**	**+ + +**
Htt-25Q	*hsp104*	**+ + +**	**+ w− −**	**+ + +**	**+ + +**
Htt-103Q	*hsp104*	**+ + +**	**+ − −**	**+ + +**	**+ + +**
Htt-25Q	*ssa1 ssa2*	**+ + +**	**w − −**	**+ w −**	**+ + +**
Htt-103Q	*ssa1 ssa2*	**+ + +**	**w − −**	**+ w −**	**+ + +**
Htt-25Q	pSSA1	**+ + +**	**+ w −**	**+ + +**	**+ + +**
Htt-103Q	pSSA1	**+ + +**	**+ w −**	**+ + w**	**+ + +**
Htt-25Q	pYDJ1	**+ + +**	**+w w −**	**+ + +**	**+ + +**
Htt-103Q	pYDJ1	**+ + +**	**+w w− −**	**+ w −**	**+ + w**
Htt-25Q	*sir2*	**+ + +**	**+ − −**	**+ + −**	**+ + w**
Htt-103Q	*sir2*	**+ + +**	**w− − −**	**+ w −**	**+ + −**
Htt-25Q	Diploid	**+ + +**	**+ +w −**	**+ + +**	**+ + +**
Htt-103Q	Diploid	**+ + +**	**+ − −**	**+ w w−**	**+ + +**

**Note:** Yeast strains were streaked into three successive diluted streaks of yeast cells as indicated from left to right in each panel. The growth characteristics refer to growth in each of these three successive diluted streaks of yeast cells (see Supplementary Figure 1 for an example). The first pool consisted of four to five toothpick streaks on the indicated medium from freshly grown cells pre-grown on glucose-containing medium. The second pool was made by choosing a new toothpick that was crossed through the first set of streaks to dilute the yeast before making another four to five streaks. Finally, a new toothpick was used to streak through this second group of streaks to further dilute the sample to obtain single cells by making another five or so streaks (third pool). Growth was on the appropriate selective plates either containing 2% glucose (glc) or 2% galactose (gal), as indicated. Growth was monitored one to three days after the initial streaking as indicated. ‘+’. Good growth; ‘w’. Weak growth. ‘−’. No growth. ‘+w’ and ‘w−’ refer to intermediate growths in the appropriate direction. Ability to continue to grow on galactose medium following 3 to 4 days of growth was also monitored for all strains. In this case, cells were picked from strains grown 3 to 4 days on medium containing galactose and restreaked on galactose containing medium. Only three strains failed to grow at all (indicating they were dead to begin with): wild-type cells containing Htt-103Q, cells overexpressing *YDJ1* containing Htt-103Q (indicating that *YDJ1* overexpression was unable to suppress Htt-103Q lethality), and cells containing the *ssa1*/*ssa2* deletion with Htt-103Q. In this latter case, because the *ssa1*/*ssa2* cells containing Htt-25Q grew, albeit poorly, the lethality of Htt-103Q with *ssa1*/*ssa2* may partly be attributed to pleiotropic effects of deleting these two *HSP70* genes.

**Table 2:** Yeast strains.

Yeast strain	Genotype
W303-1A	*Mata leu2-3*,*112 trp1-1 ura3-1 his3-11 can1-100*
TRY124-uT	Isogenic to W303-1A except *hsp104::TRP1 ura3::TRP1*
TRY125	Isogenic to W303-1A except *ssa1:HIS3 ssa2:LEU2*
W303-1B-*sir2*	Isogenic to W303-1A except *MATα* and *sir2::his5*^*+*^
W303-1B/pSSA1	Isogenic to W303-1B except carrying pLH101 (pSSA1) (*SSA1:LEU2*)
W303-1B/pYDJ1	Isogenic to W303-1B except carrying plasmid pRSYDH1 (pYDJ1) (*YDJ1:LEU2*)
