# Supplementary material for: Multiple discrete soluble aggregates influence polyglutamine toxicity in a Huntington’s disease model system
Source: Sci Rep. 2016 Oct 10;6:34916. doi: 10.1038/srep34916 (PMC5056504; doi:10.1038/srep34916)

Supplementary Figures

Multiple discrete soluble aggregates influence polyglutamine toxicity in a Huntington's disease model system

Wen Xi, Xin Wang, Thomas M. Laue, and Clyde L. Denis

Supplementary Figure 1. Growth of yeast on medium containing galactose. Wild-type yeast containing the indicated Htt-polyQ were streaked into three successive pools from left to right as indicated in Table 1. The plates were photographed at three days after streaking. The scoring of growth is given on the right.

Supplementary Figure 2. AU-FDS analysis of the monomeric Htt-103Q in different strain types. The strains used in each panel are the same as described in Figures 1, 3, and 4. Htt-103Q was induced with galactose for 1.5 hr in each case and AU-FDS analysis was conducted as described in Figure 1a.

Supplementary Figure 3. Raw data and residuals analysis following AU-FDS analysis of Htt-103Q. The top panels represent the raw data for the AU-FDS analysis for 200 scans. The bottom panels represent the residuals when comparing the Sedfit analysis to the actual data and displays the deviations for each data point for each scan. The x-axis is given in cm (distance from the center of the rotor). Data was taken for the analysis of Htt-103Q whose Sedfit analysis is displayed in Figure 1c. a- 24 hr; b- 6 hr.

Supplementary Figure 1

Htt-25Q-
Htt-103Q-

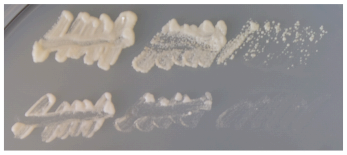

+ + +
+ w -

Supplementary Figure 2a

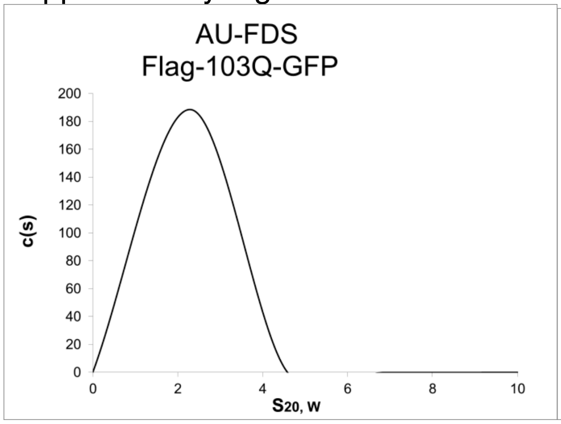

2b

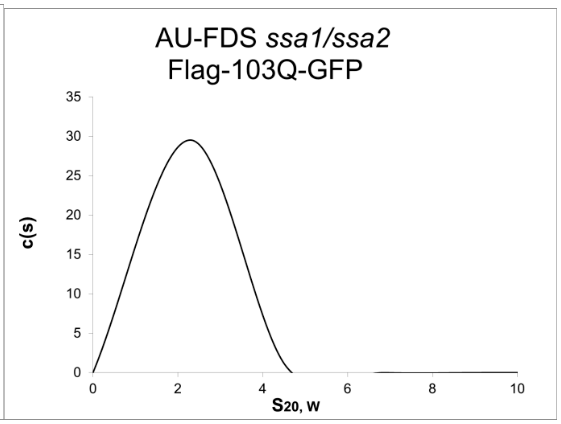

2c

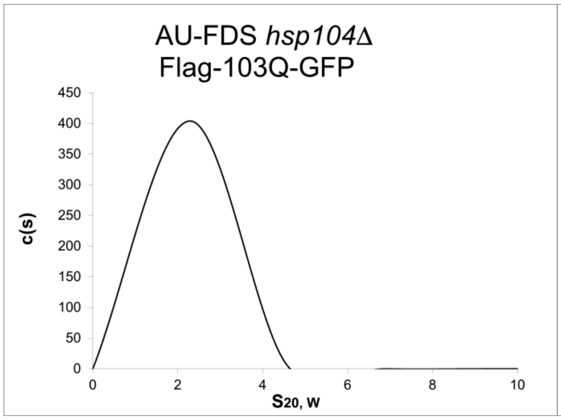

2d

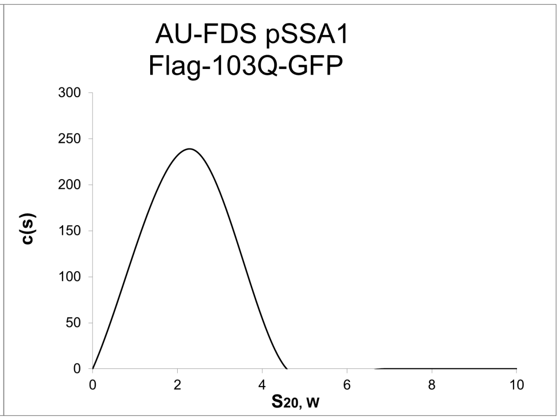

2e

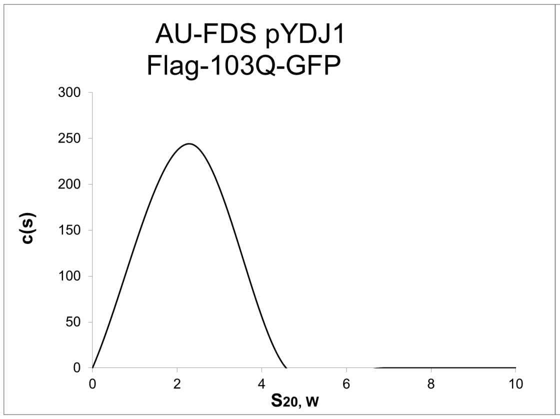

2f

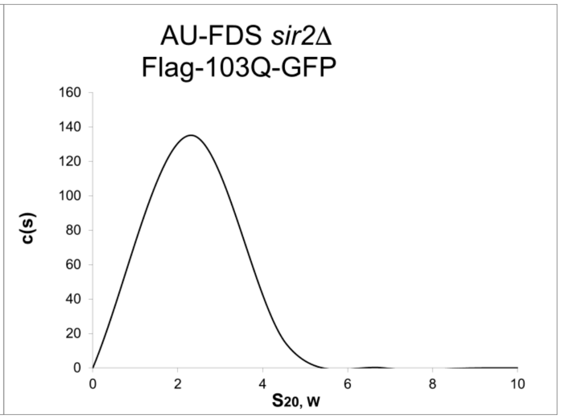

Supplementary Figure 3a

Flag-Htt-103Q-GFP 24 hr

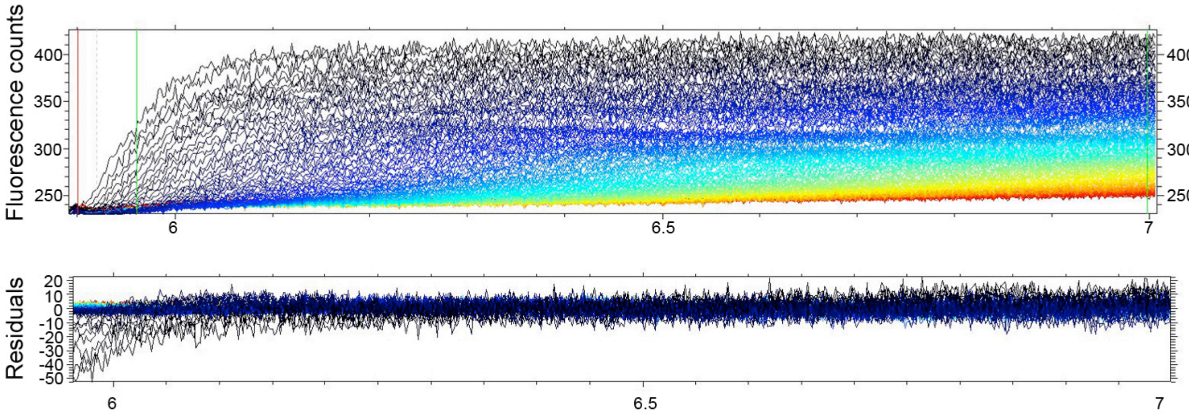

3b

Flag-Htt-103Q-GFP 6 hr

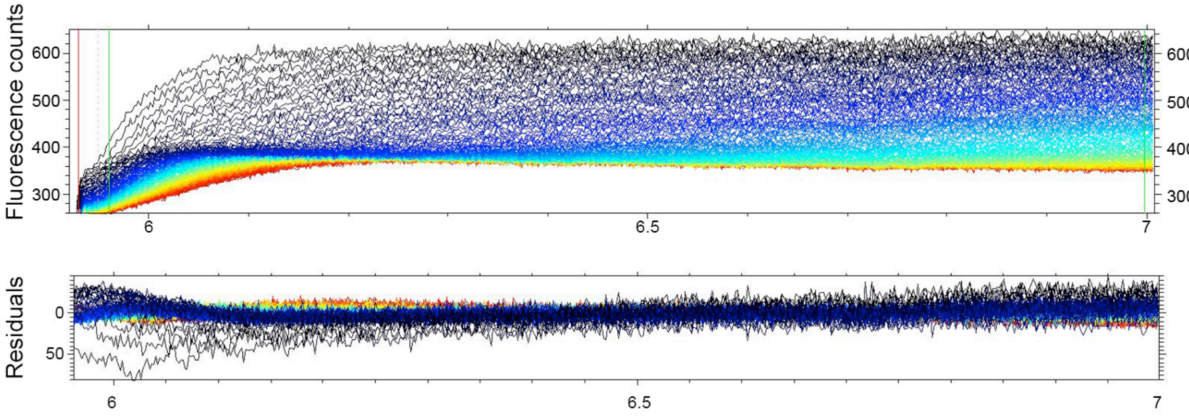

Supplement: Supplementary Information [file srep34916-s1.pdf]
